# Optimizing Fall Risk Diagnosis in Older Adults Using a Bayesian Classifier and Simulated Annealing

**DOI:** 10.3390/bioengineering11090908

**Published:** 2024-09-11

**Authors:** Enrique Hernandez-Laredo, Ángel Gabriel Estévez-Pedraza, Laura Mercedes Santiago-Fuentes, Lorena Parra-Rodríguez

**Affiliations:** 1 Tianguistenco Professional Academic Unit, Autonomous University of the State of Mexico, Tianguistenco 52640, Mexico; ehernandezl@uaemex.mx; 2 Health Science Department, Metropolitan Autonomous University, Mexico City 09310, Mexico; lmsf@xanum.uam.mx; 3 Research Department, National Institute of Geriatrics, Mexico City 10200, Mexico; lparra@inger.gob.mx

**Keywords:** fall risk classification, simulated annealing algorithm, features selection, older adults, Center of Pressure (CoP) indices

## Abstract

The aim of this study was to improve the diagnostic ability of fall risk classifiers using a Bayesian approach and the Simulated Annealing (SA) algorithm. A total of 47 features from 181 records (40 Center of Pressure (CoP) indices and 7 patient descriptive variables) were analyzed. The wrapper method of feature selection using the SA algorithm was applied to optimize the cost function based on the difference of the mean minus the standard deviation of the Area Under the Curve (AUC) of the fall risk classifiers across multiple dimensions. A stratified 60–20–20% hold-out method was used for train, test, and validation sets, respectively. The results showed that although the highest performance was observed with 31 features (0.815 ± 0.110), lower variability and higher explainability were achieved with only 15 features (0.780 ± 0.055). These findings suggest that the SA algorithm is a valuable tool for feature selection for acceptable fall risk diagnosis. This method offers an alternative or complementary resource in situations where clinical tools are difficult to apply.

## 1. Introduction

Globally, the World Health Organization (WHO) estimates that 684,000 fatal falls and 37.7 million falls serious enough to require medical attention occur annually, which involves various health problems and considerable economic costs at the public health, family, and personal levels [1]. About 35% of older adults have at least one fall per year [2,3], and this percentage increases to 32–42% for those over 70 years, making this population group one of the most vulnerable to injury or even death from a fall [1,3].

Given the relevance and implications of this public health problem in the adult population, it is important to make a correct and timely diagnosis. Balance assessment has been used to identify a possible fall risk, either through the use of technological systems [4] or by applying clinical tools based on questionnaires and standardized physical tests, such as Short Physical Performance Battery (SPPB), Timed Up and Go (TUG), Berg Balance Scale (BBS), Short Falls Efficacy Scale-International, Mini-Balance Evaluation Systems Test (Mini-BESTest), etc. [5]. However, the questionnaires have questionable accuracy and are not generalizable since they are susceptible to bias, as the evaluations are partially subjective and depend on the experience and ability of the evaluator [6]. Likewise, asking the fallers questions about the accidents they have had causes anxiety and stress due to the negative memories that are triggered, and at other times, they do not remember the fall or the number of falls [7,8]. These limitations can be reduced by using affordable technologies such as force platforms [9] and its low-cost alternatives [10,11]. These platforms allow for a quantitative study called stabilometry [12], from which Center of Pressure (CoP) indices can be obtained that allow for the characterization of the body sway by metrics and graphs [13,14].

The use of artificial intelligence techniques has made it possible to generate predictive or diagnostic models for balance alterations and/or fall risk based on sociodemographic, anthropometric, and CoP indices [15,16,17,18,19,20,21,22]. Specifically, it has been observed that Machine Learning algorithms based on Bayesian [22] and Decision Tree [15,23,24] classifiers, and Multi-Layer Perceptron [20] perform better in assessing fall risk compared with other techniques. On the other hand, Deep Learning techniques, particularly Neural Networks, are innovative methods that offer superior accuracy compared to traditional approaches [25]. However, these techniques often present challenges in interpretability, making it difficult to explain the studied phenomenon based on the input features. Furthermore, their performance may be compromised when trained on limited datasets [26,27]. This limitation is particularly relevant in the field of static stabilometry, where available data sources are often scarce [28].

Predicting an infrequent future event like falls is inherently challenging [28], so it is necessary to optimize feature selection to improve the performance of Machine Learning models [20] and provide a better explanation of which CoP indices best describe fall risk, as even with numerous research, it has been impossible to reach a consensus [29,30]. As such, this paper presents the Bayesian classification technique in combination with the heuristic approach of Simulated Annealing (SA) for feature selection to increase the diagnostic prediction of fall risk classifiers using human balance data from a sample of older adults. The current work could contribute to the production of an optimal computational model capable of predicting fall risk from quick stabilometric assessment.

## 2. Materials and Methods

### 2.1. Subjects and Preprocessing

For this study, a “public data set of human balance evaluations” database was used [31]. This includes information on 116 females and 47 males, aged 18 to 85 years. The participants were assessed repeatedly three times to obtain their stabilometric data using a force platform (OPT400600-1000; AMTI, Watertown, MA, USA), and their Short Falls Efficacy Scale-International (Short FES-I) scores were registered. Additionally, the dataset includes details such as sex, age, height, weight, body mass index (BMI), fall history, foot length, and polypharmacy.

Only information from older adults aged 60 years or older were used, who were labeled as Fall Risk if they recorded ≥1 fall in the previous 12 months and/or were rated as being of high concern in the Short FES-I. From each subject, 40 CoP indices were calculated according to Prieto [14]. To balance the dataset concerning the number of records per class, only the first set of repeated tests was selected for the Non-Fall Risk class, while for the Fall Risk class, 3 repeated tests were selected.

The CoP indices, age (years), weight (kilograms), height (centimeters), BMI (kilograms/meters^2^), and foot length (centimeters) were also used as continuous variables, polypharmacy as a discrete variable, while sex was used as a dichotomic variable (man or woman).

### 2.2. Bayesian Classifier

#### 2.2.1. Statistical Analysis

A descriptive analysis was performed. Continuous and discrete variables are presented as means and standard deviations, and sex as a number and percentage. The normality of the continuous variables was assessed using Kolmogorov–Smirnov test. Comparisons of Fall Risk versus Non-Fall Risk individuals were estimated through a T-test for parametric variables, a Mann–Whitney test for non-parametric variables, and a χ^2^ test for categorical variables. The predictive validity of a Fall Risk for all continuous and discrete variables was assessed using the Hosmer–Lemeshow Goodness of Fit test and the Area Under the Curve (AUC).

#### 2.2.2. Model Architecture

A Bayesian classifier was used to generate a fall risk model. According to Bayes’ theorem, the probability of belonging to the Fall Risk class ($P_{FR}$) is given by Equation (1):

(1) $$P_{FR}=P\times \left(\frac{1}{{\left(2\pi \right)}^{\frac{k}{2}}\times {\left|S\right|}^{\frac{1}{2}}}\right)\times e^{-\frac{1}{2}{\left(\bar{X}-\mu \right)}^{\prime }\times \frac{\left(\bar{X}-\mu \right)}{S}}$$

where P denotes the a priori probability of the classes (equiprobability between classes), μ is the mean value of the class in the feature space, S is the covariance matrix of the features, $\bar{X}$ is the feature vector, and k is the number of features. On the other hand, the probability of the class Non-Fall Risk ($P_{NFR}$) is given by the complementary probability of $P_{FR}$, which is $P_{NFR}=1-P_{FR}$. Therefore, the classifier prediction rule is given by Equation (2):

(2) $$\left\{\;\;\begin{array}{c} if\;P_{FR}>\;P_{NFR}\;\;\;\;\;\;\;\;\;\;\;\;\;\;\;\;\;\;\;\;\;\;\;\;\;\;\;\;\;\;\;Fall\;Risk\;\;\;\;\\ \;\;\;\;else\;\;\;\;\;\;\;\;\;\;\;\;\;\;\;\;\;\;\;\;\;\;\;\;\;\;\;\;\;\;\;\;\;\;\;\;\;\;\;\;\;\;\text{Non-}Fall\;Risk \end{array}\right.$$

All features’ values were standardized to a zero mean and unit variance so that they are dimensionless and have the same scale. The Bayesian classifier was coded and executed in a script of MATLAB^®^ version 2024A. For more details about the scripts, please refer to the link for the public repository on GitHub.

### 2.3. Feature Selection by the Simulated Annealing Algorithm

For the feature selection task, the SA algorithm was used to optimize the performance of the Bayesian classifier. In that sense, the problem was represented through an array with n available elements (n = Bayesian classifier number dimensions); to assign n, random indices of m features are available (m = total numbers of features). The initial solution was composed by 4 patient descriptive variables (sex, BMI, age, polypharmacy) [3,16] and 7 CoP indices (total length ML, total length AP, 95% conf. ellipse area, mean velocity, mean velocity-AP, mean frequency, and RMS distance), which have been shown to be associated with the fall risk in older adults [16,32,33,34,35]. For dimensions greater than 11 features, the initial solution was represented by the optimal feature combination from the SA optimization of the previous dimension and the addition of a random feature.

The cost function was integrated as the difference of the mean and standard deviation of the AUC of the train, test, and validation sets. On the other hand, for initial parameters, an initial temperature (T) of 0.5979 was calculated using an initial acceptance probability of 0.9 according to [36]. A stop temperature (Tmin) of 0.0232 [37], geometric cooling with an additive constant of 0.82 [36,38], and an adaptive steady state (Lk) with 30 iterations were used.

The original SA algorithm [39] was modified by adding two improvements. First, the cost function was penalized with a value equal to 0 when the sensitivity or specificity of the train, test, or validation set was less than 0.6. Second, the result of the cost function of each SA iteration was stored in a vector, with the purpose of finding the maximum value of the cost function at the end of all SA iterations. Algorithm 1 shows the pseudocode used.
**Algorithm 1: Feature selection algorithm based on simulated annealing****Input: Training dataset ** **Output: Optimal Feature Combination = best_features**1. T = 0.5979 2. Tmin = 0.0232 3. Lk = 30 4. Initial solution is declared 5. C0 = the function cost value of initial solution 6. i = 1                %% number of iterations 7. n = 11                %% n = dimensions 8. Cp = 0                %% function cost value of current solution 9. do while (T > Tmin): 10.    Generate a n-dimension random solution array 11.    Training Bayesian classifier 12.    Calculate the Bayesian classifier’s AUC for the train, test and validation sets. 13.     if ((sensibility or specificity) < 0.6): 14.         Cost_function [i] = 0 15.     else: 16.         Cost_function [i] = mean (AUC_train, AUC_test, AUC_validation) −                   std (AUC_train, AUC_test, AUC_validation) 17.     Cp = max (Cost_function) 18.     DeltaE = Cp − C0 19.     if (DeltaE >= 0): 20.          C0 = Cp 21.          features [i] = last n-dimension random solution array 22.     elseif exp(DeltaE/(T)) > rand(1,1): 23.          C0 = Cp 24.          features [i] = last n-dimension random solution array 25.     k = k + 1 26.     T = T *× 0.82 27.     Lk = Lk + Lk × (1 − exp(−1)) 28. best_features [n] = features (find (max (Cost_function)) 29. n = n + 1 30. Restart pseudocode

### 2.4. Validation Strategies and Evaluation Metrics

The most used validation method with stabilometric datasets has been the 80–20% hold-out method [15,20,23], and to ensure a better comparison, this method was selected. However, to decrease the probability of bias, the data were divided into the train, test, and validation sets, corresponding to 60%, 20%, and 20%, respectively [40], using the stratified hold-out method based on the fall risk label. The sensitivity, specificity, and AUC metrics were used to evaluate the performance of the Bayesian classifier’s optimal feature combination.

To assess the robustness of the top five feature combinations with the highest AUC, 150 new training sets were generated using the bootstrap aggregation technique from the original set. This approach enabled the construction of an ensemble learning model composed of 150 Bayesian classifiers, with the objective of analyzing in detail the impact of the optimal features through the performance of the mean AUC for fall risk diagnosis.

In addition, a univariate logistic regression model was generated for each CoP index, and its AUC was compared with the performance of the Bayesian classifier. These models were also made in MATLAB^®^ version 2024A.

## 3. Results

Information from 76 individuals was included in the study. The mean age of these participants was 71.31 ± 6.47 years, 78.94% of the sample was women, and 38.15% presented fall risk conditions. Due to the balance of the data described in Section 2.1, a total of 181 stabilometric assessments were included, of which 94 records corresponded to the Non-Fall Risk class and 87 to the Fall Risk class. Features such as sex, foot length, 50% power frequency-RD, 95% power frequency-RD, 50% power frequency-AP, total power-ML, 95% power frequency-ML, centroidal frequency-RD, frequency dispersion-AP, and frequency dispersion-AP showed significant differences between the Non-Fall Risk and Fall Risk groups. The description of general participant characteristics and statistical analysis of the CoP indices are shown in Table 1 and Table A1, respectively.

The CoP index with the best level of predictive validity according to its AUC is frequency dispersion-AP (AUC = 0.591). Table 2 shows the Top 5 CoP indices with the highest AUC and the full results are shown in Table A1.

**Figure 1:**
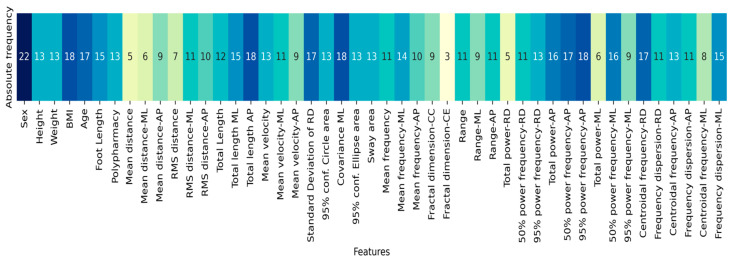
Absolute frequencies of the features selected by SA through all dimensions (n = 11 to 35). The colors refer to a gradient bar associated with the frequency of use of the features.

The SA algorithm was executed to identify the optimal feature combination, beginning with n = 11 (refer to Section 2.3). The process continued until adding more features no longer resulted in a decrease in the cost function for at least three consecutive dimensions. It was observed that after incorporating n = 32 features, the performance of the classifier began to decline (see the full content in Table A2 and the dictionary features in Table A3). Through all the iterations, sex, BMI, total length-AP, covariance-ML, and 95% power frequency-AP were the most frequent in feature selection, as shown in Figure 1.

**Figure 2:**
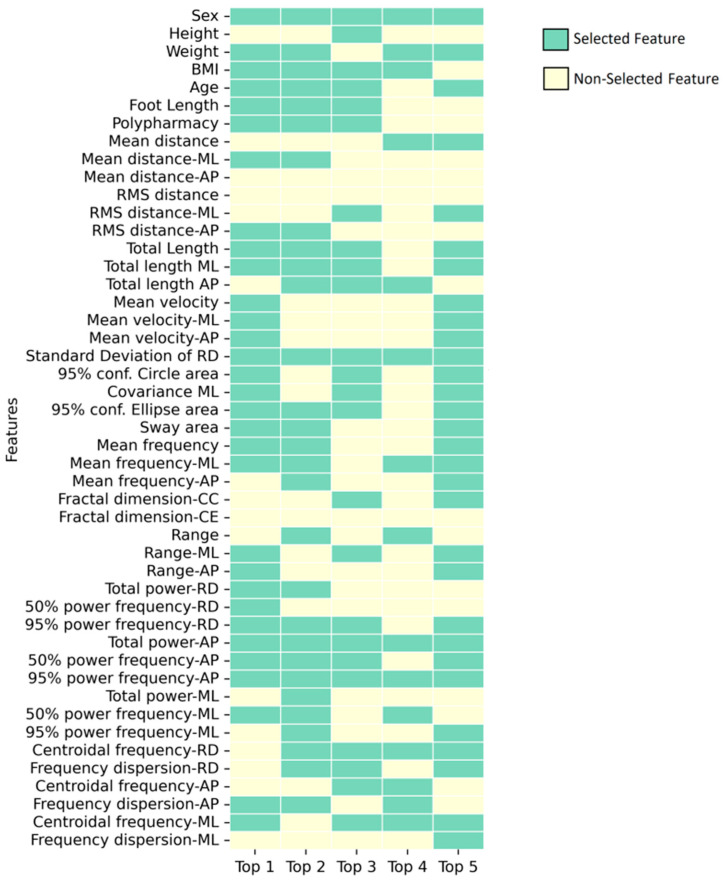
Set of features that integrate the best-performance results in feature selection.

Table 3 shows the optimal Bayesian classification models obtained using feature combinations selected by the SA algorithm. The 31-feature model (Top 1) demonstrated the highest mean AUC of 0.815 ± 0.110 for hold-out validation, though this value decreased by 8% under bootstrap aggregation validation. Conversely, the 15-feature model (Top 4) exhibited the lowest variability between sets at 0.780 ± 0.055. These features maintained their robustness more effectively, showing only a 0.7% decrease. Figure 2 illustrates the selected features comprising these top-performing classifiers.

Features such as standard deviation RD, total power AP, and sex consistently appear in all selected optimal combinations. Regarding the sex variable, it was necessary to study its possible influence given the difference in the proportion of women with respect to men in the study sample. Therefore, a mean difference analysis was performed (see results in Table 4) showing that 8 of the 14 predictor variables show a statistically significant difference between sexes.

This finding suggests that the disproportionality in the sample could introduce a bias in the generalization of the classifier’s results. However, it is pertinent to note that it has previously been suggested [41] that sex could be a relevant predictor to characterize the fall risk. In the context of the Bayesian paradigm, the conditional and marginal probabilities associated with sex could significantly contribute to the precise discrimination of fall risk classes. Nevertheless, to study the true influence of sex, it is necessary to increase the dataset heterogeneously, which underscores the importance of generating new public stabilometric datasets in biomedical research.

**Figure 3:**
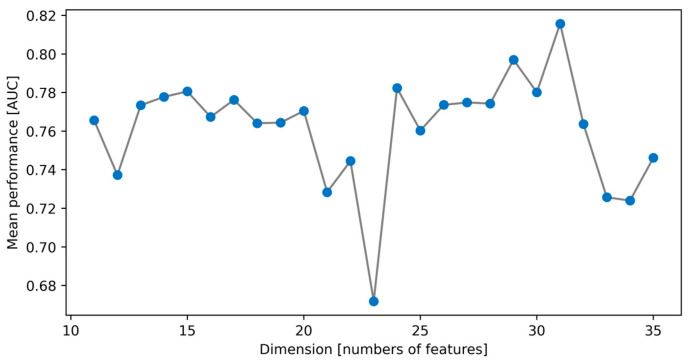
Performance of the SA algorithm based on the AUC value with respect to the dimension size.

On the other hand, Figure 3 shows the performance of the SA algorithm of the AUC mean value concerning the size of the dimensions, where no general trend is observed. This is confirmed by a correlation coefficient value of −0.083. However, the performance of the classifier was inversely correlated with the solution space, with a value of −0.303.

Moreover, the univariate logistic regression models generated for each feature presented a maximum performance for the centroidal frequency-RD CoP index, with AUC’s mean and standard deviation of 0.623 ± 0.107 for train, test, and validation sets. The complete results of the logistic regressions are available in Table A4.

## 4. Discussion

There are clinical tools that are able to predict the fall risk with the help of expert evaluators’ judgments based on extensive questionnaires to which elderly patients often do not know how to respond with certainty, and may also involve the execution of physical tests that may generate stress or fear, so previous factors alter the reliability of the results [42,43,44,45,46]. On the other hand, the use of stabilometry allows for a CoP index calculation that provides quantitative data to obtain more objective results, which, in combination with patient descriptive variables and heuristic search methods, can be useful for fall risk prediction based on computational classifier models.

The predictive capacity of classifiers based on Machine Learning benefits from feature selection, which aims at extracting the most explanatory data of the phenomenon to be predicted, and eliminating irrelevant and redundant data to reduce the dimensionality (number of features to be used) of the classifiers [47]. SA is a metaheuristic search algorithm analogously inspired by the statistical physics of heating and cooling annealing processes in metals, which can find an optimal cost function value in a large solution space. Its performance and relative ease of application have made it one of the most popular techniques for solving combinatorial problems, including feature selection [39,48,49].

Feature selection methods can be divided into filter, wrapper, and embedded methods. Filter methods perform the selection based on statistical tests such as correlations, goodness of fit, significance of coefficients, etc. On the other hand, wrapper methods select the best features by optimizing the performance of a previously chosen classification algorithm, as in the case of the Bayesian classifier optimized by SA. On the contrary, in embedded methods, feature selection is integrated in the classifier algorithm, since during the training step, its parameters are adjusted by determining the importance of each feature to produce the best diagnostic capacity [47]. Previous findings suggest that wrapper methods perform best in identifying fall risk using Machine Learning and/or statistical models [20].

This study used 47 features, of which 7 were related to participant information and 40 CoP indices, including time, frequency, and hybrid domain metrics. These were used to generate classification models based on Bayesian techniques optimized by SA, which were subsequently compared with feature selection techniques based on filter methods and univariate logistic regression models.

The best performance of the univariate logistic regression models was the centroidal frequency-RD index, which matched the selection of the Hosmer–Lemeshow goodness-of-fit methods and the mean difference test; however, its performance was poor (maximum mean AUC and standard deviation of 0.623 ± 0.107 for the training, test, and validation sets). Comparatively, the SA algorithm showed the ability to automatically identify the set of descriptor characteristics for fall risk, maximizing the diagnostic capability. Although the highest performance was presented when the algorithm selected 31 characteristics (Top 1), the results that presented less variability in the phenomenon to be predicted were given when the algorithm selected 15 characteristics (Top 4). Among these selection proposals, there was a difference of 0.035 between the diagnostic capabilities given by their AUC means.

Compared to previous studies, the predictive model proposed in Top 1 demonstrates an AUC performance improvement of at least 6.5%. Table 5 provides a detailed comparison with other works that have used static stabilometry to classify fall risk through computational or statistical methods.

Features such as sex, weight, BMI, mean distance, total length AP, standard deviation of RD, mean frequency-ML, range, total power-AP, 95% power frequency-AP, 50% power frequency-ML, centroidal frequency-RD, centroidal frequency-AP, frequency dispersion-AP, and centroidal frequency-ML compose the optimal combination (Top 4). Of these, only the features sex, weight, BMI, and total length-AP were included in the initial proposed solution based on the state of the art. This demonstrates the ability of the SA algorithm to overcome local optima by selecting features that maximize the cost function.

On the other hand, in our previous findings [51], the range, total power-AP, and standard deviation of RD indices were included among the 10 CoP indices with the highest AUC for identifying balance alterations in older adults with a high prevalence of poor physical performance identifying the optimal cut-off point, while the total power-AP, 95% power frequency-AP, and centroidal frequency-AP indices were associated with the prediction of balance alterations in healthy older adults [16]. This supports 9 of the 15 characteristics selected by the SA algorithm, and with the inclusion of the 6 complementary ones, new evidence is provided for the understanding of the fall risk phenomenon in older adults. Furthermore, the current research suggests that frequency and hybrid CoP indices have equal or better descriptive power than time domain indices. However, their use is not as widespread in the state of the art, since due to the computational power, they need to be calculated, and most commercial systems are limited to providing CoP indicators in the time domain.

In other findings, an inverse correlation was observed between the size of the search space and the ability to select an optimal combination of the SA algorithm. In the present problem, the maximum search space was given by 5.38258 × 10^11^ of possible combinations corresponding to 21 dimensions, but as shown in Figure 3, as the SA algorithm approached this maximum and the number of dimensions increased, its performance decreased. This trend continued only up to 24 dimensions (Top 3), where the solution space was reduced to 3.53697 × 10^11^. The performance of the cost function continued to improve as features were added to the classifier, from 24 up to 31. However, although the solution space kept decreasing for higher dimensions, the performance of the Bayesian classifier was affected. This suggests the occurrence of the so-called “curse of dimensionality” starting from 31 or more features in this dataset.

A limitation of the present study was the use of CoP indices derived only from stabilometric tests under conditions of a firm surface and open eyes; however, this dataset was analyzed because it comes from a test that is simpler and faster to perform and may be generalizable not only to people of different ages, but also with different cognitive and physical abilities. In this context, to avoid a disproportionate increase in the solution space affecting the performance of the SA algorithm, the number of features was limited to the 47 commonly analyzed in the stabilometric domain. Based on the performance observed in the Top 4, it would be important to highlight that future studies could incorporate new nonlinear-type experimental features and apply advanced feature extraction techniques, such as Deep Learning, Genetic Programming, and Codebook-based approaches, which have been shown to perform well in other biomedical areas, such as gait analysis [27] and heart rate variability [54], among others. Another limitation of the study lies in the data sample analyzed, since it is relatively small and has a bias influenced by the predominance of the female ratio in the sample under study, with only 21.06% corresponding to information from men.

The observation of statistically significant sex differences in Top 4 predictor variables underscores the need to apply sex-stratified analyses in future research, provided that a more complete dataset is available. Such stratification could reveal sex-specific patterns that would otherwise be hidden in general analyses. Furthermore, these potential approaches could substantially improve both the predictive accuracy and clinical utility of fall risk assessment tools.

## 5. Conclusions

The results suggest that the SA algorithm is a useful tool to perform feature selection in Bayesian classifiers for the diagnosis of fall risk from CoP indices and patient descriptive variables. This is advantageous because it provides an alternative or complementary and generalized resource with an acceptable level of fall risk assessment for people for whom the physical activities involved in clinical tools may be challenging.

## Figures and Tables

**Table 1:** Description of general participant characteristics by the fall risk group.

	Total	Non-Fall Risk	Fall Risk	*p*-Value Means Difference Test
	*n* = 76	*n* = 47	*n* = 29	
Sex [women] *n* (%)	60 (78.94)	33 (70.21)	27 (93.10)	0.017 *
Age [years]	71.3 ± 6.4	71.7 ± 6.5	70.6 ± 6.3	0.486
Height [cm]	157.2 ± 8.1	158.2 ± 9.1	155.5 ± 5.9	0.124
Weight [kg]	63.1 ± 8.4	63.6 ± 8.2	62.2 ± 8.6	0.477
BMI [kg/m^2^]	25.5 ± 2.9	25.4 ± 2.9	25.6 ± 2.9	0.760
Foot length [cm]	22.6 ± 1.3	22.9 ± 1.2	22.0 ± 1.3	0.006 *
Polypharmacy	2.3 ± 1.6	2.3 ± 1.4	2.3 ± 1.8	0.707
Fall in the last year	0.9 ± 5.9	-	2.4 ± 9.5	-

* *p*-value < 0.05.

**Table 2:** Statistical analysis of the CoP indices with the best level of predictive validity according to their AUC.

CoP Index	Total	Non-Fall Risk	Fall Risk	KS Test	MD Test	HL Test	AUC (95% CI)
	*n* = 181	*n* = 94	*n* = 87	*p*-Value	*p*-Value	*p*-Value	
Frequency dispersion-AP [-]	7.27 ± 1.06	7.13 ± 1.09	7.42 ± 1.01	0.000 *	0.034 *	0.400	0.591 (0.508–0.674)
Total power-ML [mm^2^/Hz]	63.14 ± 114.63	45.52 ± 32.64	82.17 ± 160.14	0.000 *	0.048 *	0.996	0.585 (0.501–0.668)
Total power-RD [mm^2^/Hz]	32.63 ± 56.53	25.7 ± 18.25	40.12 ± 78.86	0.000 *	0.097	0.908	0.571 (0.487–0.655)
Range-ML [mm]	27.29 ± 13.28	25.66 ± 9.35	29.06 ± 16.39	0.000 *	0.119	0.029 *	0.567 (0.482–0.651)
Range [mm]	28.50 ± 13.39	26.97 ± 9.75	30.15 ± 16.34	0.000 *	0.146	0.276	0.562 (0.478–0.647)

*n* = sample size, KS = Kolmogorov–Smirnov, MD = mean difference, HL = Hosmer–Lemeshow, CI = confidence interval, * *p*-value < 0.05.

**Table 3:** List of best-performance results in feature selection.

	Top	*n*	Train			Test			Validation			Train–Test–Validation
			SE	SP	AUC	SE	SP	AUC	SE	SP	AUC	AUC (Mean ± Std)
Hold-out	1	31	0.92	0.96	0.94	0.72	0.78	0.75	0.70	0.78	0.74	0.815 ± 0.110
	2	29	0.92	0.91	0.91	0.72	0.68	0.70	0.64	0.89	0.77	0.797 ± 0.109
	3	24	0.88	0.91	0.89	0.66	0.73	0.70	0.70	0.78	0.74	0.782 ± 0.102
	4	15	0.80	0.85	0.83	0.94	0.63	0.78	0.70	0.73	0.72	0.780 ± 0.055
	5	30	0.92	0.80	0.86	0.83	0.63	0.73	0.64	0.84	0.74	0.780 ± 0.072
Bootstrap	1	31	0.94	0.98	0.96	0.33	0.84	0.58	0.33	0.84	0.65	0.734 ± 0.200
	2	29	0.90	1.00	0.95	0.16	0.89	0.53	0.16	0.89	0.58	0.690 ± 0.228
	3	24	0.94	0.96	0.95	0.38	0.84	0.61	0.38	0.84	0.53	0.702 ± 0.220
	4	15	0.82	0.85	0.84	0.77	0.68	0.73	0.77	0.82	0.85	0.773 ± 0.059
	5	30	0.92	0.89	0.90	0.50	0.73	0.61	0.50	0.73	0.56	0.698 ± 0.183

*n* = features dimension, SE = sensitivity, SP = specificity, AUC = Area Under the Curve.

**Table 4:** Descriptive analysis of the Top 4 predictor variables by sex.

	Total	Male	Female	*p*-Value Means Difference Test
	*n* = 181	*n* = 34	*n* = 147	
Weight [kg]	62.99 ± 8.4	67.89 ± 7.05	61.86 ± 8.3	0.000 *
BMI [kg/m^2^]	25.55 ± 2.9	24.3 ± 1.89	25.83 ± 3.02	0.000 *
Mean distance [mm]	5.17 ± 2.45	6.87 ± 3.87	4.78 ± 1.79	0.004 *
Total length AP [mm]	324.71 ± 158.25	411.47 ± 215.79	304.64 ± 134.88	0.009 *
Standard deviation of RD [mm]	2.95 ± 1.48	3.93 ± 2.49	2.72 ± 1.01	0.009 *
Mean frequency-ML [Hz]	4.55 ± 1.81	4.1 ± 1.22	4.65 ± 1.92	0.040 *
Range [mm]	28.5 ± 13.39	37.35 ± 23.01	26.45 ± 8.89	0.010 *
Total power-AP [mm^2^/Hz]	22.43 ± 19.49	35.03 ± 24.25	19.51 ± 17.03	0.001 *
95% power frequency-AP [Hz]	9.83 ± 2.5	9.47 ± 2.98	9.91 ± 2.38	0.418
50% power frequency-AP [Hz]	2.66 ± 1.96	2.51 ± 2.08	2.69 ± 1.94	0.458
Centroidal frequency-RD [Hz]	7.08 ± 2.05	6.56 ± 1.55	7.2 ± 2.13	0.101
Centroidal frequency-AP [Hz]	5.42 ± 1.57	5.21 ± 1.83	5.47 ± 1.51	0.390
Frequency dispersion-ML [-]	5.88 ± 1.55	5.4 ± 1.7	5.98 ± 1.5	0.847
Centroidal frequency-ML [Hz]	7.4 ± 0.89	7.43 ± 0.91	7.39 ± 0.89	0.074

* *p*-value < 0.05.

**Table 5:** State-of-the-art performance of classifiers for fall risk detection, balance alteration, and fall history.

Work (Year)	Technology	Stabilometric Test	Dataset	Sample Size	Pre-Processing	Algorithm	Label	Validation Method	Performance
Top 1 (This work)	Force platform (OPT400600-1000) 100 Hz	Static test with open eyes	[26]	76 older adults	Compute CoP indices	BC and SA	Fall risk (FH + FES score)	60–20–20 hold-out	AUC: 0.815 SE: 0.783 SP: 0.847
Top 4 (This work)	Force platform (OPT400600-1000) 100 Hz	Static test with open eyes	[26]	76 older adults	Compute CoP indices	BC and SA	Fall risk (FH + Short FES-I)	60–20–20 hold-out	AUC: 0.780 SE: 0.818 SP: 0.741
[23] (2021)	Force platform (OPT400600-1000) 100 Hz	Static test with open and close eyes on soft and hard surface	[26]	76 older adults	Empirical Mode DeComposition, and compute CoP indices	RF	Fall risk (FH + Short FES-I e)	80–20 hold-out	SE: 0.760 SP: 0.860 ACC: 0.820
[15] (2016)	Wii Balance Board 25 Hz	Static test with open and close eyes	Own	80 older adults	Compute CoP indices	Raking Forest	FH	70–30 hold-out	AUC: 0.750
[20] (2019)	Force platform (OPT400600-1000) 100 Hz	Static test with open and close eyes on soft and hard surface	[26]	76 older adults	Compute CoP indices	MLP SVM NB K-NN and Feature selection	Fall risk (FH + Short FES-I)	80–20 hold-out	AUC: 0.710 ACC: 0.800
[50] (2018)	Force platform (OPT400600-1000) 100 Hz	Static test with open and close eyes on soft and hard surface	[26]	163 people between 18 and 85 years old	Compute CoP indices	K-NN DTs MLP NB RF SVM	Fall risk (HF + MiniBEST)	10-Fold	ACC: 0.649
[24] (2021)	Force platform (AccuSway) 120 Hz	Static test with open and close eyes	Own	126 older women with osteoporosis	Compute CoP indices, and data balancing	NB SVM AdaBoost K-NN	FH	10-Fold	SE: 0.810 SP: 0.190
[17] (2016)	Force platform (Advenced Mechanical Technology) 100 Hz	Static test with open and close eyes	Own	76 older adults	Compute CoP indices	LR	FH	None	AUC: 0.900
[51] (2022)	Wii Balance Board 50 Hz	Static test with open and close eyes	Own	46 older adults	Compute CoP indices	LR	Balance deficit (4-stage balance)	None	AUC: 0.770 SE: 0.930 SP: 0.620
[52] (2013)	Force platform (Tecnobody) 20 Hz	Static test with open and close eyes	Own	100 older adults	Compute CoP indices	LR	FH	None	SE: 0.880 SP: 0.670
[21] (2018)	Force platform (EMG system do Brasil) 100 Hz	Unipodal static test	Own	170 older adults	Compute CoP indices	ROC	FH	None	AUC: 0.720 SE: 0.660 SP: 0.680
[16] (2021)	Wii Balance Board 50 Hz	Static test with open and close eyes	Own	497 older adults	Compute CoP indices	LR	Balance alteration (4-stage balance)	None	AUC: 0.710 SE: 0.490 SP: 0.830
[19] (2015)	Wii Balance Board	Static test with open eyes	Own	73 older adults	Compute CoP indices	LR	FH	None	AUC: 0.71
[18] (2017)	Wii Balance Board 100 Hz	Static test with open and close eyes	Own	100 older adults	Compute CoP indices	Discriminant analysis	FH	None	SE: 0.710 SP: 0.570
[53] (2020)	Force platform (SmartScale-Zibro) 60 Hz	Static test with open eyes	Own	412 older adults	Compute CoP indices	ROC	FH	None	AUC: 0.640 SE: 0.640 SP: 0.590

For algorithm: BC = Bayesian classifier, SA = Simulated Annealing, RF = Random Forest, MLP = Multi-Layer Perceptron, SVM = Support Vector Machine, NB = Naïve Bayes, KNN = K-Nearest Neighbor, DTs = Decision Trees, LR = Logistic Regression, ROC = Receiver Operating Characteristic analysis. For labels: FH = fall history, Short FES-I = Short Falls Efficacy Scale-International, MiniBEST = Mini-Balance Evaluation Systems Test. For performance: SE = sensitivity, SP = specificity, AUC = Area Under the Curve, ACC = accuracy.

## Data Availability

Databases are anonymized and available as Supplementary Material.

## Supplementary Materials

Supporting information about the database, result tables, and source code can be downloaded from the public repository (https://github.com/enriquehdez98/Fall-Risk-Diagnosis-) on GitHub.

## Appendix A

**Table A1:** Statistical analysis of the CoP indices.

CoP Index	Total	Non-Fall Risk	Fall Risk	KS Test	MD Test	HL Test	AUC (95% CI)
	*n* = 181	*n* = 94	*n* = 87	*p*-Value	*p*-Value	*p*-Value	
Mean distance [mm]	5.16 ± 2.45	4.95 ± 2.02	5.39 ± 2.83	0.000 *	0.322	0.828	0.542 (0.457–0.627)
Mean distance-ML [mm]	4.00 ± 2.15	3.84 ± 1.83	4.17 ± 2.44	0.000 *	0.339	0.515	0.541 (0.456–0.625)
Mean distance-AP [mm]	2.46 ± 1.18	2.35 ± 0.98	2.57 ± 1.36	0.000 *	0.430	0.824	0.534 (0.449–0.618)
RMS distance [mm]	5.96 ± 2.83	5.71 ± 2.29	6.23 ± 3.31	0.000 *	0.318	0.503	0.543 (0.458–0.627)
RMS distance-ML mm]	5.00 ± 2.65	4.78 ± 2.16	5.24 ± 3.09	0.000 *	0.303	0.43	0.544 (0.459–0.628)
RMS distance-AP [mm]	3.08 ± 1.42	2.97 ± 1.24	3.2 ± 1.59	0.000 *	0.448	0.448	0.532 (0.447–0.617)
Total Length [mm]	713.92 ± 333.48	694.21 ± 269.03	735.22 ± 391.93	0.000 *	0.607	0.116	0.477 (0.391–0.564)
Total length ML [mm]	563.81 ± 272.52	536.17 ± 217.65	593.67 ± 320.13	0.000 *	0.943	0.015 *	0.503 (0.414–0.591)
Total length AP [mm]	324.71 ± 158.24	330.34 ± 141.11	318.62 ± 175.51	0.000 *	0.161	0.026 *	0.439 (0.355–0.524)
Mean velocity [mm/s]	11.89 ± 5.55	11.57 ± 4.48	12.25 ± 6.53	0.000 *	0.607	0.116	0.477 (0.391–0.564)
Mean velocity-ML [mm/s]	9.39 ± 4.54	8.93 ± 3.62	9.89 ± 5.33	0.000 *	0.943	0.015 *	0.503 (0.414–0.591)
Mean velocity-AP [mm/s]	5.41 ± 2.63	5.5 ± 2.35	5.31 ± 2.92	0.000 *	0.161	0.026 *	0.439 (0.355–0.524)
Standard deviation of RD [mm]	2.94 ± 1.47	2.82 ± 1.13	3.08 ± 1.77	0.000 *	0.267	0.465	0.547 (0.463–0.632)
95% conf. Circle area [mm^2^]	38.79 ± 55.95	33.49 ± 31.05	44.52 ± 73.8	0.000 *	0.276	0.586	0.547 (0.462–0.631)
Covariance ML [mm^2^]	0.01 ± 0.87	0.11 ± 0.84	-0.09 ± 0.9	0.000 *	0.214	0.848	0.446 (0.362–0.53)
95% conf. Ellipse area [mm^2^]	31.48 ± 37.62	27.33 ± 23.57	35.97 ± 48.19	0.000 *	0.214	0.15	0.553 (0.468–0.638)
Sway area [mm^2^/s]	2.01 ± 2.35	1.78 ± 1.46	2.26 ± 3.02	0.000 *	0.619	0.46	0.521 (0.435–0.607)
Mean frequency [Hz]	3.90 ± 1.43	3.97 ± 1.37	3.82 ± 1.51	0.000 *	0.181	0.299	0.442 (0.357–0.527)
Mean frequency-ML [Hz]	4.54 ± 1.81	4.55 ± 1.75	4.53 ± 1.89	0.000 *	0.718	0.597	0.484 (0.399–0.569)
Mean frequency-AP [Hz]	4.19 ± 1.70	4.39 ± 1.72	3.96 ± 1.67	0.000 *	0.079	0.427	0.424 (0.34–0.508)
Fractal dimension-CC [-]	17.04 ± 1.18	17.12 ± 1.15	16.95 ± 1.22	0.027 *	0.205	0.028 *	0.445 (0.36–0.53)
Fractal dimension-CE [-]	17.29 ± 1.13	17.38 ± 1.05	17.2 ± 1.21	0.025 *	0.092	0.217	0.427 (0.342–0.512)
Range [mm]	28.50 ± 13.39	26.97 ± 9.75	30.15 ± 16.34	0.000 *	0.146	0.276	0.562 (0.478–0.647)
Range-ML [mm]	27.29 ± 13.28	25.66 ± 9.35	29.06 ± 16.39	0.000 *	0.119	0.029 *	0.567 (0.482–0.651)
Range-AP [mm]	16.82 ± 7.23	16.51 ± 6.89	17.15 ± 7.61	0.000 *	0.723	0.248	0.515 (0.43–0.6)
Total power-RD [mm^2^/Hz]	32.63 ± 56.53	25.7 ± 18.25	40.12 ± 78.86	0.000 *	0.097	0.908	0.571 (0.487–0.655)
50% power frequency-RD [Hz]	3.21 ± 1.86	3.45 ± 1.88	2.94 ± 1.8	0.000 *	0.023 *	0.78	0.402 (0.319–0.485)
95% power frequency-RD [Hz]	14.10 ± 4.05	14.61 ± 3.75	13.54 ± 4.29	0.002 *	0.032 *	0.044 *	0.407 (0.323–0.492)
Total power-AP [mm^2^/Hz]	22.42 ± 19.49	21.6 ± 18.89	23.31 ± 20.19	0.000 *	0.727	0.452	0.515 (0.43–0.599)
50% power frequency-AP [Hz]	2.65 ± 1.96	2.91 ± 2.09	2.38 ± 1.77	0.000 *	0.039 *	0.077	0.411 (0.328–0.494)
95% power frequency-AP [Hz]	9.82 ± 2.50	9.91 ± 2.44	9.73 ± 2.57	0.200	0.639	0.191	0.48 (0.395–0.565)
Total power-ML [mm^2^/Hz]	63.14 ± 114.63	45.52 ± 32.64	82.17 ± 160.14	0.000 *	0.048 *	0.996	0.585 (0.501–0.668)
50% power frequency-ML [Hz]	2.61 ± 1.80	2.8 ± 1.84	2.41 ± 1.74	0.000 *	0.056	0.425	0.417 (0.334–0.501)
95% power frequency-ML [Hz]	10.99 ± 2.76	11.38 ± 2.57	10.57 ± 2.9	0.200	0.048 *	0.24	0.442 (0.357–0.526)
Centroidal frequency-RD [Hz]	7.08 ± 2.04	7.37 ± 1.94	6.75 ± 2.11	0.028 *	0.017 *	0.172	0.396 (0.313–0.48)
Frequency dispersion-RD [-]	7.32 ± 0.61	7.27 ± 0.65	7.38 ± 0.56	0.005 *	0.188	0.628	0.556 (0.472–0.64)
Centroidal frequency-AP [Hz]	5.41 ± 1.57	5.61 ± 1.6	5.2 ± 1.51	0.013 *	0.090	0.054	0.427 (0.343–0.51)
Frequency dispersion-AP [-]	7.27 ± 1.06	7.13 ± 1.09	7.42 ± 1.01	0.000 *	0.034 *	0.400	0.591 (0.508–0.674)
Centroidal frequency-ML [Hz]	5.87 ± 1.54	6.12 ± 1.45	5.6 ± 1.6	0.200	0.022 *	0.081	0.418 (0.334–0.502)
Frequency dispersion-ML [-]	7.39 ± 0.89	7.35 ± 0.89	7.44 ± 0.89	0.000 *	0.371	0.088	0.538 (0.453–0.623)

*n* = sample size, KS = Kolmogorov–Smirnov, MD = mean difference, HL = Hosmer–Lemeshow, CI = confidence interval, * *p*-value < 0.05

**Table A2:** Bayesian classifier performance results using Simulated Annealing.

n	Combination of Optimal Features	Train			Test			Validation		
		SE	SP	AUC	SE	SP	AUC	SE	SP	AUC
11	1, 2, 15, 19, 20, 21, 36, 37, 43, 44, 45	0.769	0.75	0.759	0.888	0.736	0.812	0.764	0.684	0.724
12	1, 2, 4, 10, 16, 17, 23, 30, 34, 37, 42, 47	0.75	0.821	0.785	0.777	0.631	0.704	0.705	0.736	0.721
13	1, 4, 5, 16, 17, 18, 22, 29, 30, 37, 42, 44, 47	0.865	0.75	0.807	0.833	0.631	0.732	0.823	0.736	0.78
14	1, 3, 4, 8, 12, 16, 18, 22, 35, 36, 37, 42, 44, 47	0.788	0.803	0.796	0.888	0.684	0.786	0.764	0.736	0.75
15	1, 3, 4, 8, 16, 20, 26, 30, 36, 38, 40, 42, 44, 45, 46	0.807	0.857	0.832	0.944	0.631	0.788	0.705	0.736	0.721
16	1, 2, 4, 6, 7, 12, 13, 16, 17, 20, 22, 27, 32, 38, 42, 43	0.846	0.803	0.824	0.722	0.736	0.729	0.705	0.789	0.747
17	1, 2, 4, 5, 7, 9, 13, 17, 22, 27, 30, 35, 37, 38, 40, 43, 47	0.884	0.821	0.853	0.666	0.736	0.701	0.705	0.842	0.773
18	1, 3, 4, 6, 7, 8, 11, 19, 20, 24, 27, 28, 30, 34, 36, 38, 40, 42	0.711	0.857	0.784	0.777	0.736	0.757	0.764	0.736	0.75
19	2, 4, 5, 6, 7, 9, 10, 11, 13, 14, 16, 20, 32, 33, 34, 36, 40, 42, 47	0.788	0.892	0.84	0.666	0.684	0.675	0.764	0.789	0.777
20	1, 3, 4, 6, 7, 10, 11, 14, 15, 18, 20, 22, 24, 31, 34, 35, 37, 42, 44, 47	1	0.696	0.848	0.722	0.631	0.676	0.941	0.631	0.786
21	1, 2, 4, 5, 6, 11, 14, 15, 16, 20, 21, 22, 23, 24, 35, 36, 37, 39, 40, 41, 45	0.826	0.75	0.788	0.777	0.631	0.704	0.647	0.736	0.691
22	1, 2, 3, 5, 12, 16, 20, 21, 22, 25, 26, 27, 28, 30, 31, 34, 38, 40, 44, 45, 46, 47	0.903	0.714	0.809	0.722	0.684	0.703	0.705	0.736	0.721
23	1, 2, 6, 9, 10, 11, 13, 14, 15, 16, 18, 23, 26, 29, 32, 34, 38, 39, 40, 41, 42, 45, 47	0.634	0.714	0.674	0.666	0.684	0.675	0.647	0.684	0.665
24	1, 2, 4, 5, 6, 7, 12, 14, 15, 16, 20, 21, 22, 23, 28, 31, 35, 36, 37, 38, 42, 43, 44, 46	0.884	0.91	0.897	0.666	0.736	0.701	0.705	0.789	0.747
25	1, 3, 5, 10, 12, 16, 17, 18, 19, 20, 21, 22, 23, 24, 25, 26, 28, 32, 35, 36, 38, 41, 42, 44, 45	0.826	0.839	0.833	0.722	0.789	0.755	0.647	0.736	0.691
26	1, 2, 3, 5, 7, 9, 10, 12, 15, 18, 20, 21, 22, 24, 25, 26, 27, 31, 32, 34, 37, 38, 40, 44, 46, 47	0.98	0.75	0.865	0.777	0.631	0.704	0.764	0.736	0.75
27	2, 3, 5, 6, 12, 13, 15, 16, 17, 20, 21, 22, 23, 24, 25, 26, 27, 30, 33, 35, 36, 37, 38, 40, 42, 43, 47	0.846	0.839	0.842	0.666	0.736	0.701	0.823	0.736	0.78
28	1, 2, 4, 5, 6, 7, 10, 12, 13, 14, 15, 16, 17, 22, 25, 26, 27, 28, 31, 32, 33, 35, 37, 38, 40, 41, 42, 43	0.961	0.785	0.873	0.777	0.631	0.704	0.647	0.842	0.744
29	1, 3, 4, 5, 6, 7, 9, 13, 14, 15, 16, 20, 23, 24, 25, 26, 27, 30, 33, 35, 36, 37, 38, 39, 40, 41, 42, 43, 45	0.923	0.91	0.916	0.722	0.684	0.703	0.647	0.894	0.77
30	1, 3, 5, 8, 12, 14, 15, 17, 18, 19, 20, 21, 22, 23, 24, 25, 26, 27, 28, 31, 32, 35, 36, 37, 38, 41, 42, 43, 46, 47	0.923	0.803	0.863	0.833	0.631	0.732	0.647	0.842	0.744
31	1, 3, 4, 5, 6, 7, 9, 13, 14, 15, 17, 18, 19, 20, 21, 22, 23, 24, 25, 26, 31, 32, 33, 34, 35, 36, 37, 38, 40, 45, 46	0.923	0.964	0.943	0.722	0.789	0.755	0.705	0.789	0.747
32	2, 4, 5, 6, 7, 8, 11, 13, 15, 16, 17, 18, 19, 21, 22, 23, 24, 25, 26, 28, 32, 36, 37, 38, 40, 41, 42, 43, 44, 45, 46, 47	1	0.785	0.892	0.722	0.684	0.703	0.705	0.684	0.695
33	1, 3, 4, 5, 6, 7, 10, 12, 14, 15, 16, 17, 18, 19, 21, 22, 23, 24, 25, 26, 27, 30, 34, 35, 36, 37, 38, 39, 40, 41, 44, 45, 47	0.769	0.785	0.777	0.777	0.631	0.704	0.705	0.684	0.695
34	1, 4, 5, 6, 10, 11, 12, 14, 15, 16, 17, 19, 20, 21, 22, 23, 24, 25, 26, 28, 30, 31, 32, 34, 36, 37, 38, 39, 40, 42, 43, 44, 45, 47	0.807	0.857	0.832	0.611	0.684	0.647	0.647	0.736	0.691
35	1, 3, 4, 5, 6, 7, 13, 14, 15, 16, 17, 18, 19, 20, 21, 22, 23, 24, 26, 28, 29, 30, 31, 32, 34, 35, 36, 38, 39, 40, 41, 43, 44, 46, 47	0.865	0.821	0.843	0.722	0.684	0.703	0.647	0.736	0.691

*n* = features dimension, SE = sensitivity, SP = specificity, AUC = Area Under the Curve.

**Table A3:** Dictionary Bayesian classifier performance results using Simulated Annealing.

**Feature**	**Label Meaning**
1	Sex
2	Height [cm]
3	Weight [kg]
4	BMI [kg/m^2^]
5	Age [years]
6	Foot length [cm]
7	Polypharmacy
8	Mean distance [mm]
9	Mean distance-ML [mm]
10	Mean distance-AP [mm]
11	RMS distance [mm]
12	RMS distance-ML [mm]
13	RMS distance-AP [mm]
14	Total Length [mm]
15	Total length ML [mm]
16	Total length AP [mm]
17	Mean velocity [mm/s]
18	Mean velocity-ML [mm/s]
19	Mean velocity-AP [mm/s]
20	Standard deviation of RD [mm]
21	95% conf. circle area [mm^2^]
22	Covariance ML [mm^2^]
23	95% conf. ellipse area [mm^2^]
24	Sway area [mm^2^/s]
25	Mean frequency [Hz]
26	Mean frequency-ML [Hz]
27	Mean frequency-AP [Hz]
28	Fractal dimension-CC [-]
29	Fractal dimension-CE [-]
30	Range [mm]
31	Range-ML [mm]
32	Range-AP [mm]
33	Total power-RD [mm^2^/Hz]
34	50% power frequency-RD [Hz]
35	95% power frequency-RD [Hz]
36	Total power-AP [mm^2^/Hz]
37	50% power frequency-AP [Hz]
38	95% power frequency-AP [Hz]
39	Total power-ML [mm^2^/Hz]
40	50% power frequency-ML [Hz]
41	95% power frequency-ML [Hz]
42	Centroidal frequency-RD [Hz]
43	Frequency dispersion-RD [-]
44	Centroidal frequency-AP [Hz]
45	Frequency dispersion-AP [-]
46	Centroidal frequency-ML [Hz]
47	Frequency dispersion-ML [-]

**Table A4:** Logistic regression performance.

Feature	Train			Test			Validation		
	SE	SP	AUC	SE	SP	AUC	SE	SP	AUC
Sex	0.903	0.303	0.603	1.000	0.210	0.605	0.941	0.368	0.654
Height [cm]	0.538	0.553	0.546	0.333	0.473	0.403	0.294	0.526	0.410
Weight [kg]	0.480	0.642	0.561	0.222	0.368	0.295	0.411	0.684	0.547
BMI [kg/m^2^]	0	1	0.5	0	1	0.5	0	1	0.5
Age [years]	0.250	0.821	0.535	0.388	0.789	0.589	0.235	0.894	0.565
Foot length [cm]	0.557	0.66	0.609	0.444	0.526	0.485	0.470	0.789	0.630
Polypharmacy	0.423	0.75	0.586	0.333	0.684	0.508	0.470	0.578	0.524
Mean distance [mm]	0.230	0.821	0.526	0.222	0.842	0.532	0.058	0.789	0.424
Mean distance-ML [mm]	0.250	0.839	0.544	0.111	0.789	0.450	0.294	0.789	0.541
Mean distance-AP [mm]	0.192	0.857	0.524	0.277	0.894	0.586	0.058	0.789	0.424
RMS distance [mm]	0.250	0.839	0.544	0.222	0.842	0.532	0.058	0.789	0.424
RMS distance-ML [mm]	0.250	0.821	0.535	0.111	0.789	0.450	0.235	0.789	0.512
RMS distance-AP [mm]	0.192	0.875	0.533	0.222	0.947	0.584	0.058	0.842	0.450
Total length [mm]	0.307	0.839	0.573	0.166	0.736	0.451	0.117	0.789	0.453
Total length ML [mm]	0.423	0.839	0.631	0.166	0.736	0.451	0.176	0.842	0.509
Total length AP [mm]	0	1	0.5	0	1	0.5	0	1	0.5
Mean velocity [mm/s]	0.307	0.839	0.573	0.166	0.736	0.451	0.117	0.789	0.453
Mean velocity-ML [mm/s]	0.423	0.839	0.631	0.166	0.736	0.451	0.176	0.842	0.509
Mean velocity-AP [mm/s]	0	1	0.5	0	1	0.5	0	1	0.5
Standard deviation of RD [mm]	0.134	0.821	0.478	0.222	0.842	0.532	0.176	0.842	0.509
95% conf. circle area [mm^2^]	0.096	0.857	0.476	0.166	0.842	0.504	0.058	0.842	0.45
Covariance ML [mm^2^]	0.269	0.803	0.536	0.222	0.789	0.505	0.294	0.789	0.541
95% conf. ellipse area [mm^2^]	0.134	0.892	0.513	0.222	0.947	0.584	0.058	0.842	0.450
Sway area [mm^2^/s]	0.230	0.857	0.543	0.111	0.947	0.529	0.176	0.894	0.535
Mean frequency [Hz]	0.250	0.839	0.544	0.166	0.684	0.425	0.176	0.789	0.482
Mean frequency-ML [Hz]	0.326	0.767	0.547	0.333	0.473	0.403	0.176	0.736	0.456
Mean frequency-AP [Hz]	0.365	0.660	0.513	0.388	0.789	0.589	0.529	0.631	0.58
Fractal dimension-CC [-]	0.057	0.982	0.519	0	0.947	0.473	0	0.947	0.473
Fractal dimension-CE [-]	0.076	0.982	0.529	0	0.947	0.473	0	0.894	0.447
Range [mm]	0.230	0.821	0.526	0.166	0.789	0.478	0.294	0.842	0.568
Range-ML [mm]	0.326	0.803	0.565	0.222	0.789	0.505	0.235	0.842	0.538
Range-AP [mm]	0.019	0.982	0.5	0	1	0.5	0	1	0.5
Total power-RD [mm^2^/Hz]	0.211	0.857	0.534	0.111	0.894	0.502	0.176	0.894	0.535
50% power frequency-RD [Hz]	0.480	0.678	0.579	0.444	0.631	0.538	0.647	0.473	0.560
95% power frequency-RD [Hz]	0.250	0.892	0.571	0.333	0.894	0.614	0.470	0.842	0.656
Total power-AP [mm^2^/Hz]	0.250	0.857	0.553	0.055	0.947	0.501	0.058	0.947	0.503
50% power frequency-AP [Hz]	0.576	0.553	0.565	0.611	0.315	0.463	0.764	0.526	0.645
95% power frequency-AP [Hz]	0.019	1	0.509	0	1	0.5	0	0.947	0.473
Total power-ML [mm^2^/Hz]	0.269	0.892	0.581	0.166	0.894	0.53	0.176	0.789	0.482
50% power frequency-ML [Hz]	0.480	0.642	0.561	0.444	0.736	0.59	0.588	0.526	0.557
95% power frequency-ML [Hz]	0.365	0.714	0.539	0.333	0.631	0.482	0.411	0.684	0.547
Centroidal frequency-RD [Hz]	0.384	0.732	0.558	0.444	0.684	0.564	0.705	0.789	0.747
Frequency dispersion-RD [-]	0.250	0.732	0.491	0.444	0.789	0.616	0.529	0.631	0.58
Centroidal frequency-AP [Hz]	0.423	0.714	0.568	0.444	0.578	0.511	0.588	0.684	0.636
Frequency dispersion-AP [-]	0.538	0.553	0.546	0.555	0.473	0.514	0.647	0.421	0.534
Centroidal frequency-ML [Hz]	0.403	0.678	0.541	0.388	0.684	0.536	0.529	0.578	0.554
Frequency dispersion-ML [-]	0.192	0.875	0.533	0.222	0.947	0.584	0.294	0.789	0.541

SE = sensitivity, SP = specificity, AUC = Area Under the Curve.
